# Social, economic, and environmental factors influencing the basic reproduction number of COVID-19 across countries

**DOI:** 10.1371/journal.pone.0252373

**Published:** 2021-06-09

**Authors:** Jude Dzevela Kong, Eden Tekwa, Sarah A. Gignoux-Wolfsohn

**Affiliations:** 1 Centre for Diseases Modeling (CDM), York University, Toronto, ON, Canada; 2 Department of Mathematics and Statistics, York University, Toronto, ON, Canada; 3 Department of Ecology, Evolution, and Natural Resources, Rutgers University, New Brunswick, NJ, United States of America; 4 Department of Ecology and Evolutionary Biology, Princeton University, Princeton, NJ, United States of America; 5 Department of Zoology, University of British Columbia, BC, Canada; 6 Smithsonian Environmental Research Center, Edgewater, MD, United States of America; Universita degli Studi del Molise, ITALY

## Abstract

**Objective:**

To assess whether the basic reproduction number (*R*_*0*_) of COVID-19 is different across countries and what national-level demographic, social, and environmental factors other than interventions characterize initial vulnerability to the virus.

**Methods:**

We fit logistic growth curves to reported daily case numbers, up to the first epidemic peak, for 58 countries for which 16 explanatory covariates are available. This fitting has been shown to robustly estimate *R*_*0*_ from the specified period. We then use a generalized additive model (GAM) to discern both linear and nonlinear effects, and include 5 random effect covariates to account for potential differences in testing and reporting that can bias the estimated *R*_*0*_.

**Findings:**

We found that the mean R0 is 1.70 (S.D. 0.57), with a range between 1.10 (Ghana) and 3.52 (South Korea). We identified four factors—population between 20–34 years old (youth), population residing in urban agglomerates over 1 million (city), social media use to organize offline action (social media), and GINI income inequality—as having strong relationships with *R*_*0*_, across countries. An intermediate level of youth and GINI inequality are associated with high *R*_*0*_, (n-shape relationships), while high city population and high social media use are associated with high *R*_*0*_. Pollution, temperature, and humidity did not have strong relationships with *R*_*0*_ but were positive.

**Conclusion:**

Countries have different characteristics that predispose them to greater intrinsic vulnerability to COVID-19. Studies that aim to measure the effectiveness of interventions across locations should account for these baseline differences in social and demographic characteristics.

## Introduction

The COVID-19 pandemic, caused by the SARS-CoV-2 virus, has passed the first peak in the majority of countries in the world. Scientists, health officials and citizens have tried to anticipate and explain why the epidemic initially (i.e., before novel interventions) unfolded differently among countries; now we have the relevant data with sufficient global reach and temporal length to conduct statistical analyses. Existing studies that examine some of the factors that may contribute to differences among countries together are generally applied to metrics such as mortality, daily and cumulative case numbers, or effective reproduction number [1–4]. These metrics are time varying and sensitive to reporting and testing differences, and are therefore not easily comparable across countries. For instance, decreasing testing would allow the reported cases to drop, making raw case reporting incomparable across countries.

A key metric, *R*_*0*_, has the practical advantage of being reliably estimable [5] and comparable across countries even if testing and reporting rates are different, so long as these rates are either constant or change in roughly the same way over time. *R*_*0*_ is the basic reproduction number that indicates how many secondary infections are caused by an infected individual at the beginning of an epidemic [6]. Without interventions, the portion of the population that is expected to be infected or immunized before the epidemic ends would be 1-1/*R*_*0*_. For example, an *R*_*0*_ of 3 implies that ⅔ of the population would have to be infected or immunized by the end of the epidemic. *R*_*0*_ for COVID-19 has variably been estimated between 1.4 [7] and 8.9 [8], with a likely value of 2.5 [9]. Many studies either implicitly assume or are understood to imply that *R*_*0*_ is intrinsic to the infectious disease [9], but it is increasingly acknowledged that many non-interventive factors could affect heterogeneity in *R*_*0*_ among local populations or countries [10]. Interventive responses that occur during the initial exponential phase of COVID-19 can be understood as proximate causes of differences in *R*_*0*_ across populations, but ultimately they are likely pre-adaptations anchored on existing social, demographic, and environmental factors. Later interventions generally affect *R*_*e*_, the effective reproduction number at any given time during the epidemic [4].

Our goal is to use a diverse and comprehensive set of demographic, social, and environmental-climatic factors to begin explaining differences in the initial dynamics of COVID-19 across countries. The predictors are non-contemporary with COVID-19, meaning they were measured before the current epidemic began. The dependent variable is the basic reproduction number *R*_*0*_, which is derived from the maximum growth rate of COVID-19 (number of additional hosts infected per infected individual per day) within a country. *R*_*0*_ can be estimated from the beginning of epidemic curves [5]. The results in this study cannot be used to infer the eventual epidemic sizes among countries, which are still unfolding and can be very different from the initial dynamics due to novel interventions. We exclude proximal explanations of *R*_*0*_, such as enacted policies during the initial rise of COVID-19, because such explanations would contain statistical endogeneity—the initial epidemic growth may have partly caused the responses, therefore the responses cannot be simply used as predictors. Instead, our study focuses on how pre-existing country characteristics can explain the initial growth phases of COVID-19, although still without implying causation. We did not attempt to include all possibly relevant covariates because of high correlations even among a limited set, and because the limited number of countries dictate that a small subset should be preselected in order to retain sufficiently positive degrees of freedom for statistical analyses. Observed correlation between the covariates tested here and *R*_*0*_ may be caused by any number of other covariates that correlate with the identified covariates. Observed relationships should therefore be used for hypothesis generation and further investigation.

Covariates chosen belonged to seven categories: demographics, disease, economics, environment, habitat, health, and social. All of these categories have been suggested previously as possible factors for COVID-19 transmission. The most common factors previously studied were temperature [11–24], pollution [13,25–31], precipitation/humidity [24,32,33], population density [34,35], age structure [1,36,37], and population size [1,11,31]. For these and additional covariates either previously studied or only mentioned in the media, we rely on statistics measured at a national level. A review of previously found effects on initial COVID-19 epidemic rates related to *R*_*0*_ are documented in Table 1. We examined these categories simultaneously in order to better understand which group may have a larger influence on *R*_*0*_ and should therefore be investigated further at both the national and other scales. This analysis is not meant to be exhaustive or definitive, but rather to help reveal baseline epidemiological differences across countries, shape the direction of future research on COVID-19, and understand infectious disease transmission in general.

**Table 1:** Covariates and previous findings.

Category	Covariate	Previously found effects
demographics	**Youth:** Population ages 20–35 (% of total population) [38]	(+) [36,37]
		(-) [1]
demographics	**Total Pop:** Population total [38]	(+) [11,31]
		(0) [1]
disease	**Mort Resp:** Mortality rate from lower respiratory infections (per 100,000) [39]	(0) [40]
disease	**Mort Infect:** Mortality rate from infectious and parasitic diseases (per 100,000) [39]	(-) [41]
economic	**GINI:** GINI index (income inequality, 100 = high) [42]	(+) [43]
		(-) [36]
economic	**Business:** Ease of doing business index 2019 (1 = most business-friendly regulations) [44]	
environmental	**Temperature:** ^o^C January-March [45]	(-) [11–17,24]
		(+) [18–21]
		(0) [22]
		(n-shape) [23]
environmental	**Precipitation:** mm January-March [45]	(+) [32,33]
		(u-shape) [24]
environmental	**Pollution:** PM2.5 air pollution, mean annual exposure (micrograms per cubic meter) [38]	(+) [13,25–30]
		(-) [31]
habitat	**City:** Population in urban agglomerations of more than 1 million (% of total population) [38]	(+) [34,35]
habitat	**Urbanization:** Urban population (% of total population) [38]	(+) [34,35]
Health	**GHS:** Global Health Security detection index [46]	(0) [1]
Health	**Nurses:** Nurses and midwives (per 1,000 people) [38]	(-) [1]
Social	**Social Media:** Average People’s Use Of Social Media To Organize Offline Action (4 = high) [47]	(-?) [48]
		(+?) [49]
Social	**Internet Filtering:** Government Internet filtering in practice (4 = low) [47]	
Social	**Air Transport:** passengers carried per capita [38]	(+) [36]
		(0) [50]

Data sources are cited under the covariate column. Previous effects on epidemic rates are not necessarily on basic reproduction number *R*_*0*_, but rather on cumulative case load, daily cases at certain stages, or effective reproduction number. Effects on epidemic rates are recorded as positive (+), negative (-), insignificant (0), or non-monotonic (u-shape or n-shape). Effects accompanied by (?) are theoretical.

## Methods

All data and code are available on a Github repository [51].

### Estimating the basic reproduction number of COVID-19 among countries

**Fig 1:**
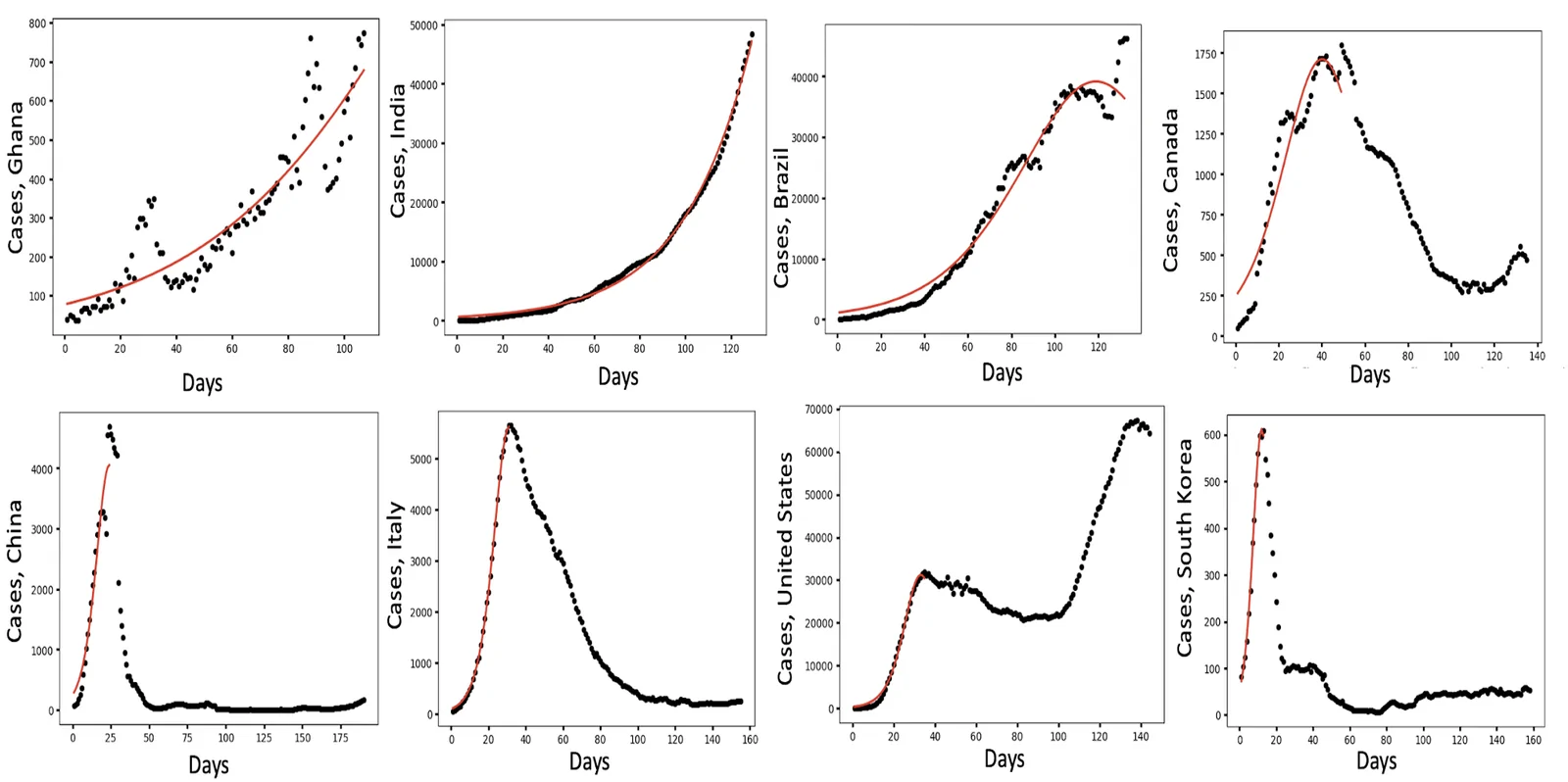
The COVID-19 daily cases. Dots represent daily cases averaged over a 7-day window, and curves are fitted based on the logistic growth model. Example countries are arranged from top left to bottom right in order of increasing basic reproduction number (*R*_*0*_).

The basic reproduction number *R*_*0*_ (the dependent variable) is given by the formula [52]:


$$R_{0}=e^{rT}$$ (1)

where *T* is the serial interval of COVID-19 (time delay between the symptom onset of a primary case and their secondary case) and *r* the initial growth rate of COVID-19. *T* has been estimated to be between 4–8 days; here we use 5.8 days [53–55]. To Estimate *r*, we fit the rate of change in cumulative cases of a logistic growth model, with parameters *r* (intrinsic growth rate) and *K* (theoretical epidemic size without intervention), to observe time series in daily confirmed cases [5,56]. The logistic growth model is superior to fitting an exponential curve to early case numbers given that case numbers do plateau in reality. In addition, the logistic growth model performs as well or better than more complicated models when confronted with data [5,57]. Mechanistic models with multiple compartments [58] and with time-dependent rates [59,60], may be more realistic for COVID-19 outbreaks that in some places exhibit multiple peaks early on, but such models contain more parameters, require much more data, and are statistically harder to infer reliably. Such complexity is also likely not necessary to describe the initial outbreaks, which appear qualitative logistic (Fig 1).

In the logistic growth model, the cumulative case number *I* is given by:


$$I(t)=\frac{K}{1+ae^{-rt}}$$ (2)

- $\frac{K}{1+a}$ is the initial number of infected persons i.e $\frac{K}{1+a}=I(0),\;a=\frac{K-I(0)}{I(0)}$
- *K* is the total number of people infected at the end of the outbreak
- *r* is the intrinsic or the maximum infection growth rate per infected host (growth rate for short)
- $\left(\frac{lnln(a)}{r},\frac{K}{2}\right)$ the point of maximum spread of SARS-CoV-2
- *r/K* is the effective between-virus competition rate, where competition is for susceptible hosts

We truncate all COVID-19 reported daily case time series [61,62] to the day with the highest daily count, because some countries have lingered near peak daily count for much longer than a logistic growth model would predict, which would pull the model peak to later than the actual date of peak incidence and thereby underestimates *r*. We manually checked each time series and ensured that the highest daily count only occurred during a first peak. We included all countries that were at least 6 days into a period with at least 30 daily cases as of July 29, 2020, after truncating at the peak. We eliminated countries whose logistic growth model *R*^*2*^ was less than 0.9. Countries were assigned to the regions of Asia-Australia, Africa, Eurasia, Europe, Middle East, North America, and South America. Eurasia included countries that simultaneously belong to both Asia and Europe, plus Ukraine and Uzbekistan due to geopolitical proximity. The Dominican Republic was assigned to the North America region, while Panama was assigned to the South America region, as these were the only Central American countries in the final list.

Some countries do not report daily, have variable reporting delays, and may have changed reporting methods resulting in dramatic spikes in cases for particular dates. To circumvent this inaccuracy in date, we used the 7-day rolling average (right aligned) for daily cases [61,62]. While this rolling average causes data from nearby dates to be autocorrelated, it should only underestimate the *p*-value of the fit but not bias the parameter estimates. *R*_*0*_ across countries were plotted using the R-package ‘rworldmap’, which uses open access data from naturalearthdata.com.

### Covariates

Next, we compiled data on predictors for each of the countries studied from seven categories (demographics, disease, economics, environmental, habitat, health, and social) from publicly available databases (Table 1).

We chose covariates that are diverse, specific, and do not obviously covary; for example, gross domestic product per capita was not used because it covaries with many other more precise covariates. In addition, we chose covariates that are comparable across countries; for example, we chose nurses per capita over doctors per capita because in many countries, nurses are the primary caregivers. For each predictor, we used the most recent available data, which ranged from 2000–2019. When appropriate, data reported in absolute numbers were divided by total population to obtain per capita figures. Data with highly skewed distributions were log-transformed and all distributions were centred and standardized before regression. Four additional covariates were examined but were eliminated through sequential variance inflation factor (VIF) analysis based on the mixed effect generalized additive model described in Section 2.3 (adapted from the ‘rms::vif’ package in RStudio1.2.5033). The goal is to reduce the collinearity of the final covariate set, so that we can make better statistical attributions to how each covariate affects *R*_*0*_. In the analysis, we eliminated the covariate with the highest VIF and iterated the elimination procedure until a representative and epidemiologically reasonable set was left (the set in Table 1). The eliminated covariates were: 1. population greater than 65 years old [38], 2. life expectancy at birth [38], 3. hospital beds per capita [38], and 4. mortality rate attributed to unsafe water, unsafe sanitation and lack of hygiene [39].

### Statistical analysis

After compiling the variables, we fitted the generalized additive model (GAM) using the ‘mgcv’ package in RStudio1.2.5033, to analyze the effects of the covariates listed in Table 1, on the *R*_*0*_ value across the globe. The covariates are standardized for effect comparisons. The main advantage of GAMs over traditional regression methods are their capability to model non-linear relationships (a common feature of many datasets) between a response variable and multiple covariates using non-parametric smoothers. The general formula of a GAM is:


$$g(\mu_{i})=\beta +\sum_{j=1}^{n}f_{j}(X_{i})+\varepsilon_{i}$$ (3)

Where *g*(*μ_i_*) is a monotonous link function relating the independent variable to the given covariates, *β* is any strictly parametric component in the model, such as intercept, *f_j_*(*X_i_*) is the variable explained by the nonparametric smoothing function, and *ε_i_* is identically and independently distributed as a normal random variable.

Two sets of analyses are performed: 1. fixed effect model on *R*_*0*_; 2. mixed effect model on *R*_*0*_, with region, the total number of days to the first 30 cases (measured from when China had her first 30th cases), gross domestic product per capita (GDP), average under-reported percentage [63], and total number of available data points as random effects. These random effects are meant to capture differences in reporting and testing standard. GDP [38] is additionally expected to correlated with many other covariates, so using it as a random effect allows us to better understand the effects of other more precise and less aggregative metrics.

There are concerns that different COVID-19 detection capabilities among nations may affect the estimated growth rates of the disease and the regression results. Some estimates of detection differences among countries have been made [64]. However, we observe that if under-reporting is constant in time within countries, then the estimated *r* and therefore *R*_*0*_ would not be affected—only *K* would be artificially depressed. On the other hand, if under-reporting is non-constant in time, then *r* would be affected [65]. For example, a country that responds strongly after the arrival of COVID-19 may ramp up testing capability, which would decrease under-reporting over time. This would cause *r* fitted to the reported case data to be an overestimate. On the other hand, if a country’s detection capability erodes over time due to a shortage of test kits or a decision to stop testing non-severe cases, then *r* would be underestimated. There are ongoing efforts to correct for these temporal biases based on delayed mortality rates [63,66], but the results are currently not credible for smaller countries with poor reporting. At this point we must rely on the reported case numbers, and use random effects to partially account for possible biases.

We use the *anova()* function in R to compare the candidate models and see which one provides the best parsimonious fit of the data. Because these models differ in the use of the random variables, ANVOA will test whether or not including random effects leads to a significant improvement over using just the given covariates without any random variables. For goodness of fits test, we use a chi-squared test.

## Results

### Basic reproduction number of COVID-19 among countries

**Fig 2:**
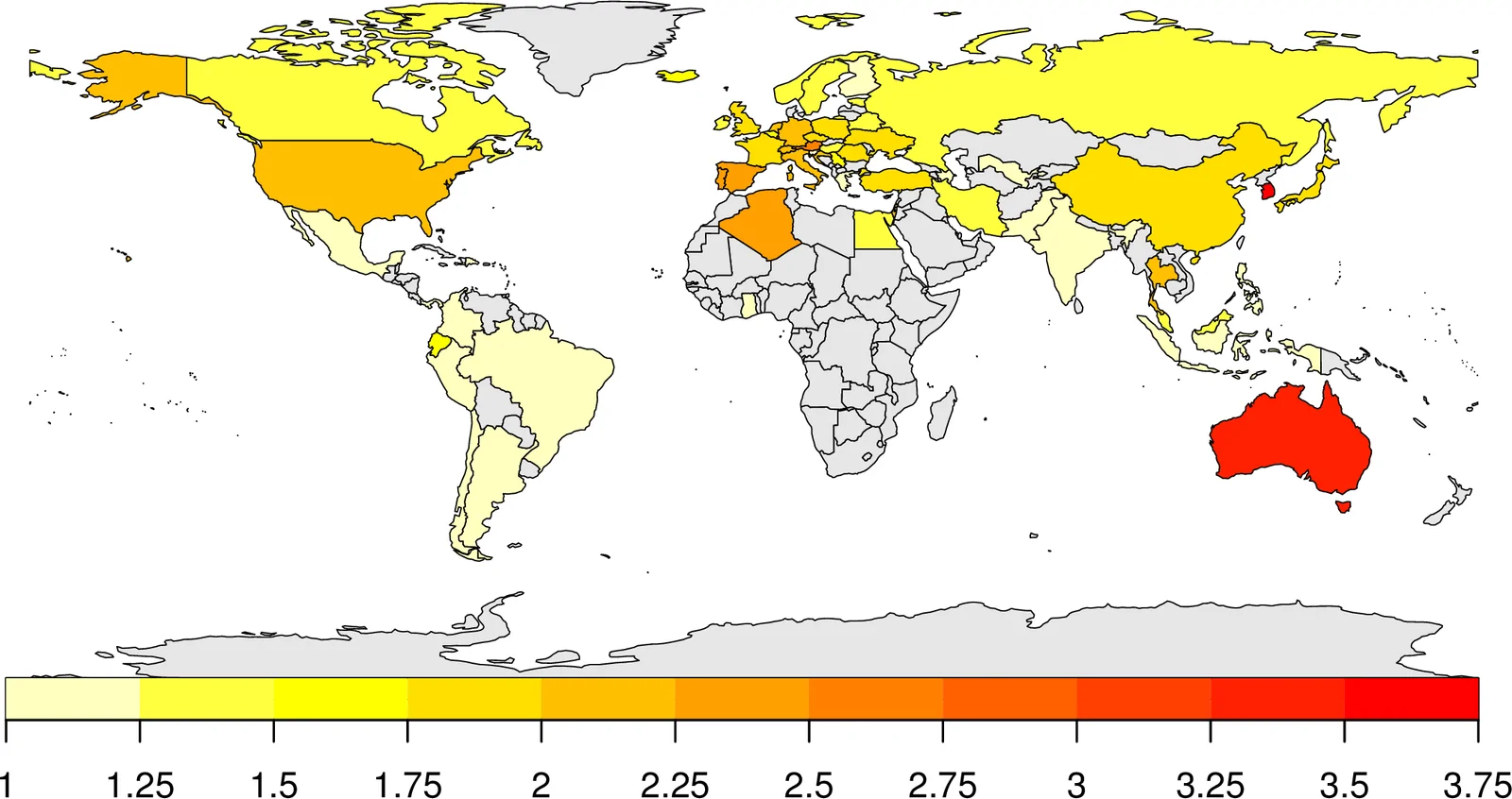
Estimated basic reproduction numbers (*R*_*0*_) for countries across the globe. Gray countries are not included in our analysis.

Fig 1 and S1–S4 Figs show growth curves fit to observed time series in daily confirmed cases across countries. Fig 2 and S1 Table summarize the estimated basic reproduction number *R*_*0*_ across countries. For the countries considered, *R*_*0*_ is highest in South Korea, Australia and Luxemburg, with 3.52, 3.35 and 3.00 and lowest in the Dominican Republic, Ghana and Indonesia with 1.10, 1.10, 1.11. Overall, the mean *R*_*0*_ is 1.70 with a standard deviation of 0.57. Belgium (1.71), Iceland (1.72), and Japan (1.79) are the closest to this mean *R*_*0*_.

### Mixed effects GAM model

**Fig 3:**
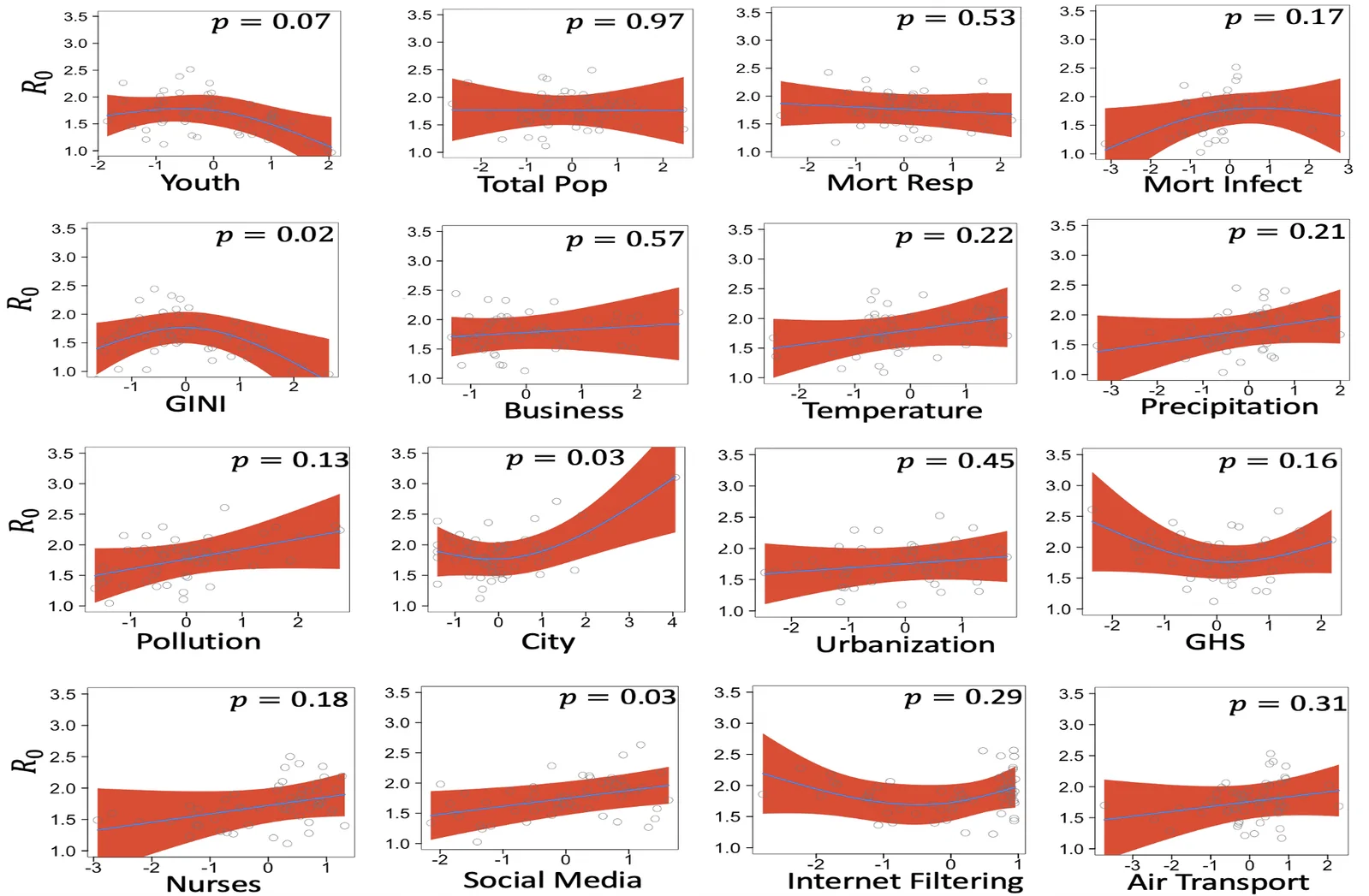
Mixed GAM derived partial effects (smoother plot) of the covariates, on *R*_*0*_. Circles are partial residuals, and red shades are 95% confidence intervals.

The explained deviance is 75.3%, this indicates that the model has a high explanatory power and predictability. The four fixed effect covariates with *p*-values below 0.1 are youth, city, social media, and GINI inequality. An intermediate value of youth (population between 20–34 years old) and GINI inequality are correlated with high *R*_*0*_. On the other hand, an intermediate level of city population (population in urban conglomerates over 1 million) is correlated with low *R*_*0*_. Finally, social media use to organize offline action is positively correlated with high *R*_*0*_ (Fig 3, S2 Table).

**Fig 4:**
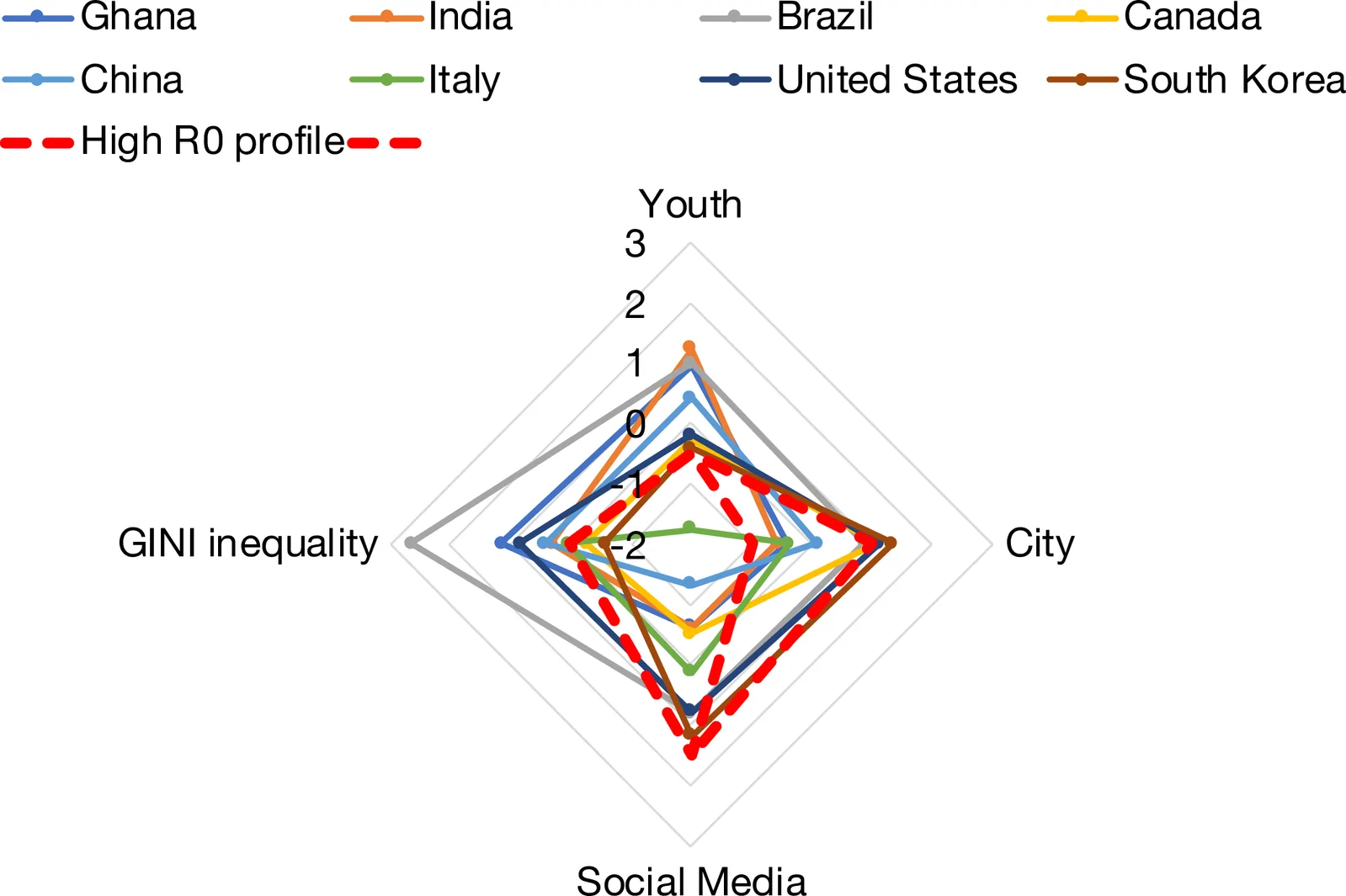
Country profiles. The four characteristics (youth, city, social media, and GINI inequality) with the lowest *p*-values in the mixed effect GAM are plotted (centred and standardized) for 8 countries representing, from top left to bottom right in the legend, increasing *R*_*0*_. Red dashed lines represent alternative high *R*_*0*_ profiles based on the mixed effects GAM model.

Fig 4 shows how eight countries covering a wide range of *R*_*0*_ are characterized by different demographic and social profiles. The profiles show that the countries’ ranking in covariate values mostly conform to the statistical trends suggested by GAM. For example, Ghana, with the nearly lowest *R*_*0*_, has a low portion of population in large urban agglomerates, relatively low social media usage, a large youth population, and a high GINI inequality index, which conform with the profile for low *R*_*0*_. South Korea and the United States, which have high *R*_*0*_ values, have a high portion of population in large urban agglomerates, a high social media usage, and an intermediate youth population, which conform with the profile for high *R*_*0*_. However, South Korea also has a relatively low GINI, while the United States has a relatively high GINI, whereas an average GINI is overall associated with the highest *R*_*0*_. This illustrates that countries with high *R*_*0*_ tend to fit the statistical high *R*_*0*_ profile in most but not all dimensions. Other countries examined, with lower *R*_*0*_, had profiles that diverge further from the statistical high *R*_*0*_ profile (Fig 4).

### Comparison with the fixed effect GAM model

For the fixed effects only model, the explained deviance is 65.5%. S5 Fig shows the effect of the covariates on *R*_*0*_, and S3 Table contains the statistical results. The ANOVA comparison of the mixed and fixed effects models shows a DF Deviance of 1.793 and *p* = 0.006. This means that adding random effects to the model lead to a significantly improved fit over the fixed effects model. Some covariates have *p*-values below 0.1 in the fixed effects model but not in the mixed effects model (temperature, internet filtering). Conversely, some covariates have lower *p*-values in the mixed effects than in the fixed effects model (city, social media). These differences illustrate that random effects are important in controlling for potential biases in the raw daily COVID-19 reporting data.

## Discussion

We found that across the globe, *R*_*0*_ (1.70±0.57 S.D.) was variable and on average slightly lower than previous estimates [8,9]. However, previous studies focussed on data from China and other countries with early epidemic onset, which our estimates show to have higher than average *R*_*0*_. We identified four factors (youth, city, social media, and GINI inequality) as having strong relationships with COVID-19 *R*_*0*_ across countries. Environmental factors, which are the most common factors previously identified (temperature [11–24], pollution [13,25–31], precipitation/humidity [24,32,33]), did not have strong relationships with *R*_*0*_ when other factors are considered simultaneously, although pollution, temperature, and humidity all have positive associations.

The positive relationship between social media usage and *R*_*0*_ observed here has not been previously found for COVID-19. The trend may be proximally caused by the propagation of false information on social media, for example in downplaying the potential danger of COVID-19, the effectiveness of masks and social distancing, or propping up conspiracy theories on the disease [49]. One study showed that more than 80% of online claims about COVID-19 were false at the beginning of the pandemic [67]. These proximal mechanisms, at least at the initial onset of COVID-19, seemed to have overridden the potential benefits of social media as an accurate information spreader that allows people to assess the true risks [48]. This result may be related to the finding that in social networks false information spreads faster than the truth [68]. In the initial stage of COVID-19, there was an information void regarding the nature of the disease and effective interventions, so it appears that false information filled an important void for people in countries where social media and reality were tightly weaved. However, social media may help slow a contagion’s spread once scientific information becomes available. Our result highlights the need to consider the dynamic role that social media plays in epidemics [69].

The quadratic relationships of youth and GINI inequality with *R*_*0*_ indicate a more complex underlying tradeoff than is previously appreciated, which was either monotonically positive [36,43] or negative [1,36]. A large youth population may confer resilience against the disease [36,37] while also increasing the transmission rate [70]; conversely an old population may be more susceptible to the disease [71,72] but exhibits a reduced transmission rate. The synergistic result is that an intermediate level of youth is related to the highest *R*_*0*_. A high GINI inequality index, referring to the amount of income inequality across a population, may mean the physical segregation of population segments and thus initially halting Covid growth across the population [36]. In contrast, a low GINI may indicate better social integrations and fewer people left at high risk exposures [43]. Therefore, an intermediate GINI is related to the highest *R*_*0*_, suggest that the hypothetical risk mechanisms—youth transmission and elder susceptibility, and social mixing and uneven risk exposures—work synergistically (rather than antagonistically) when both are present. That is, these risk mechanisms together lead to a more-than-additive increase in *R*_*0*_.

An intermediate level of city-dwelling population (population in urban conglomerates over 1 million) is related to the lowest *R*_*0*_. A high level of city dwelling is expected to increase *R*_*0*_ because of high contact rates and conforms with the main empirical trend [34]. However, it is unclear why a low level of city dwelling is also associated with a high *R*_*0*_, although the rise is relatively slight. In comparison to the quadratic effects of youth and GINI inequality, the effect of city dwelling appears close to monotonic.

Our approach has a number of limitations that should be properly acknowledged. Our analysis is based on coarse-grained country-level case data. The factors we analyzed hold across regions within a country to some extent, but it can be argued that each factor or its substitute can be measured more locally [10,73] and result in better statistical power. While we found results at the global scale that indicate real patterns, *p* values were modest. We tried to control for differences in country case reporting using random effects, but this may be insufficient. An alternative option would be to correct *R*_*0*_ estimates by modelling how temporal trends in test number, test positivity rate, and mortality affect detection, but this technique has not yet been perfected [63]. *R*_*0*_ can also be estimated using less phenomenological, more mechanistic models such as multiple-compartment (eg. susceptible-exposed-infectious-recovered-susceptible) [58,74], social network [75], or time-varying [59,60] models. However, these approaches are more data intensive and not available in many countries. As well, our analysis assumed that initial interventions specifically in response to COVID-19 were part of a country’s pre-existing characteristics, but it is possible that they were already learning from other countries even during the initial exponential phase. Other studies that desire to partition interventions from factors considered here are valid but ask a different set of questions. Our country-level analysis of *R*_*0*_ serves as a coarse grain baseline of nation’s susceptibility that future analyses can improve on with data at higher spatial resolutions. An international perspective like the one we took here can help us understand COVID-19 in a broader context, even though we sacrifice the ability to infer local causality.

We emphasize that *R*_*0*_ is not indicative of eventual outbreak sizes or the nature of subsequent waves. Given the same population, a higher *R*_*0*_ can lead to a higher outbreak size, but this does not account for intervention measures that occur after the initial epidemic growth. For instance, a high initial epidemic growth may provide a strong signal to both citizens and governments, which then may mount a stronger response to limit the outbreak size than if the initial growth were weaker. For example, South Korea and Australia had high *R*_*0*_ (3.52, 3.35) but low cumulative case numbers (490, 1073 per million on Oct 17, 2020 [61,62]). In contrast, countries such as Brazil and Peru have low *R*_*0*_ (1.18, 1.24) and yet struggle to control the epidemic (cumulative case number = 24,465, 26,156 per million on Oct 17, 2020 [61,62]). The dynamic coupling between *R*_*0*_ and response is one reason why it is harder to infer the effectiveness of intervention without taking into account how pre-existing characteristics relate to initial epidemic growth. It is reasonable to believe that early interventions are actually symptoms of pre-existing social, demographic, and environmental characteristics and are not easy to implement in other countries.

The factors influencing *R*_*0*_ identified here reflect the naive or intrinsic factors that may determine a country’s vulnerability to the novel Coronavirus. While both government and citizen interventions have since been implemented in different ways, the current study can inform both the ongoing effort to control the pandemic and efforts to anticipate and control future coronavirus epidemics. The *R*_*0*_ values calculated here serve as baseline expectations for how fast COVID-19 would spread if interventions were to be prematurely lifted, given that the percent of population susceptible to COVID-19 is still relatively low. One baseline expectation for *R*_*e*_ (effective reproductive number) without novel interventions could be $\hat{R_{e}}=\hat{R_{0}}S/N$, where *S*/*N* is the portion of the population that remains susceptible [6], and $\hat{R_{0}}$ is the model predicted *R*_*0*_ of a location given updated covariates, particularly on environmental and air transport factors that have drastically changed since the initial stage. $\hat{R_{e}}$ could then serve as a null hypothesis for analyses on whether and how much intervention has impacted the spread of COVID. Additionally, variant strains may have altered *R*_*0*_ even though the population is no longer naïve to the virus, so an updated expectation for *R*_*e*_ could take variants into account [76]. Future studies that aim to measure the effectiveness of interventions across locations should account for intrinsic factors identified here. Otherwise, interventions in countries with intrinsically low *R*_*0*_ may be mistaken as more effective than they are, while effective interventions in countries with intrinsically high *R*_*0*_ may be regrettably ignored.

## Conclusion

Scientists, health officials and citizens have routinely compared COVID-19 progressions between countries in hope of identifying risk and mitigation factors, but countries are different from each other in many more aspects than are commonly considered, making simple comparisons potentially misleading. By correlating the basic reproduction number R_0_, estimated systematically from epidemiological data, with social, economic, and environmental covariates, we found that countries differ in their intrinsic vulnerability to COVID-19 due to several characteristics. Studies that aim to measure the effectiveness of interventions on reducing the effective reproduction number could consider building location-specific null hypotheses based on model-generated expected rates as we did here.

## Supporting information

S1 Fig The COVID-19 daily cases. Dots represent daily cases averaged over a 7-day window, and curves are fitted based on the logistic growth model.
(DOCX)

S2 Fig The COVID-19 daily cases. Dots represent daily cases averaged over a 7-day window, and curves are fitted based on the logistic growth model.
(DOCX)

S3 Fig The COVID-19 daily cases. Dots represent daily cases averaged over a 7-day window, and curves are fitted based on the logistic growth model.
(DOCX)

S4 Fig The COVID-19 daily cases. Dots represent daily cases averaged over a 7-day window, and curves are fitted based on the logistic growth model.
(DOCX)

S5 Fig Fixed GAM derived effects of covariates on *R_0_* across countries. Circles are partial residuals, and red shades are 95% confidence intervals.
(DOCX)

S6 Fig Mixed GAM derived random effects on *R_0_.* Circles are partial residuals, and red shades are 95% confidence intervals.
(DOCX)

S1 Table COVID-19 intrinsic growth rates and *R_0_.* (DOCX)

S2 Table Mixed effect GAM results. An edf of 1 is equivalent to a straight line. An edf of 2 is equivalent to a quadratic curve, and so on, with higher edfs describing more wiggly curves.
(DOCX)

S3 Table Fixed effect GAM results. An edf of 1 is equivalent to a straight line. An edf of 2 is equivalent to a quadratic curve, and so on, with higher edfs describing more wiggly curves.
(DOCX)

## References

[1] Chaudhry R, Dranitsaris G, Mubashir T, Bartoszko J, Riazi S. A country level analysis measuring the impact of government actions, country preparedness and socioeconomic factors on COVID-19 mortality and related health outcomes. EClinicalMedicine. 2020;25: 100464. doi: 10.1016/j.eclinm.2020.100464 32838237 PMC7372278

[2] Martins LD, da Silva I, Batista WV, Andrade M de F, Freitas ED de, Martins JA. How socio-economic and atmospheric variables impact COVID-19 and influenza outbreaks in tropical and subtropical regions of Brazil. Environmental Research. 2020;191: 110184. doi: 10.1016/j.envres.2020.110184 32946893 PMC7492183

[3] Scarpone C, Brinkmann ST, Große T, Sonnenwald D, Fuchs M, Walker BB. A multimethod approach for county-scale geospatial analysis of emerging infectious diseases: a cross-sectional case study of COVID-19 incidence in Germany. Int J Health Geogr. 2020;19: 32. doi: 10.1186/s12942-020-00225-1 32791994 PMC7424139

[4] Li Y, Campbell H, Kulkarni D, Harpur A, Nundy M, Wang X, et al. The temporal association of introducing and lifting non-pharmaceutical interventions with the time-varying reproduction number (R) of SARS-CoV-2: a modelling study across 131 countries. The Lancet Infectious Diseases. 2020; S1473309920307854. doi: 10.1016/S1473-3099(20)30785-4 33729915 PMC7581351

[5] Ma J, Dushoff J, Bolker BM, Earn DJD. Estimating Initial Epidemic Growth Rates. Bull Math Biol. 2014;76: 245–260. doi: 10.1007/s11538-013-9918-2 24272389

[6] Ridenhour B, Kowalik JM, Shay DK. Unraveling R0: Considerations for Public Health Applications. American Journal of Public Health. 2014;104: e32–41. doi: 10.2105/AJPH.2013.301704 24328646 PMC3935673

[7] Li Q, Guan X, Wu P, Wang X, Zhou L, Tong Y, et al. Early Transmission Dynamics in Wuhan, China, of Novel Coronavirus–Infected Pneumonia. N Engl J Med. 2020;382: 1199–1207. doi: 10.1056/NEJMoa2001316 31995857 PMC7121484

[8] Sanche S, Lin YT, Xu C, Romero-Severson E, Hengartner N, Ke R. High Contagiousness and Rapid Spread of Severe Acute Respiratory Syndrome Coronavirus 2. Emerg Infect Dis. 2020;26: 1470–1477. doi: 10.3201/eid2607.200282 32255761 PMC7323562

[9] Petersen E, Koopmans M, Go U, Hamer DH, Petrosillo N, Castelli F, et al. Comparing SARS-CoV-2 with SARS-CoV and influenza pandemics. The Lancet Infectious Diseases. 2020;20: e238–e244. doi: 10.1016/S1473-3099(20)30484-9 32628905 PMC7333991

[10] Shaw J. COVID-19 May Be Much More Contagious Than We Thought. Harvard Magazine. 13 May 2020. Available: https://www.harvardmagazine.com/2020/05/r-nought

[11] Nakada LYK, Urban RC. COVID-19 pandemic: environmental and social factors influencing the spread of SARS-CoV-2 in São Paulo, Brazil. Environ Sci Pollut Res. 2020 [cited 2 Nov 2020]. doi: 10.1007/s11356-020-10930-w 32989697 PMC7521763

[12] Haque SE, Rahman M. Association between temperature, humidity, and COVID-19 outbreaks in Bangladesh. Environmental Science & Policy. 2020;114: 253–255. doi: 10.1016/j.envsci.2020.08.012 32863760 PMC7447231

[13] Lolli S, Chen Y-C, Wang S-H, Vivone G. Impact of meteorological conditions and air pollution on COVID-19 pandemic transmission in Italy. Sci Rep. 2020;10: 16213. doi: 10.1038/s41598-020-73197-8 33004925 PMC7530996

[14] Demongeot J, Flet-Berliac Y, Seligmann H. Temperature Decreases Spread Parameters of the New Covid-19 Case Dynamics. Biology. 2020;9: 94. doi: 10.3390/biology9050094 32375234 PMC7284740

[15] Sajadi MM, Habibzadeh P, Vintzileos A, Shokouhi S, Miralles-Wilhelm F, Amoroso A. Temperature, Humidity, and Latitude Analysis to Estimate Potential Spread and Seasonality of Coronavirus Disease 2019 (COVID-19). JAMA Netw Open. 2020;3: e2011834. doi: 10.1001/jamanetworkopen.2020.11834 32525550 PMC7290414

[16] Li H, Xu X-L, Dai D-W, Huang Z-Y, Ma Z, Guan Y-J. Air pollution and temperature are associated with increased COVID-19 incidence: A time series study. International Journal of Infectious Diseases. 2020;97: 278–282. doi: 10.1016/j.ijid.2020.05.076 32502664 PMC7266595

[17] Islam N, Bukhari Q, Jameel Y, Shabnam S, Erzurumluoglu AM, Siddique MA, et al. COVID-19 and climatic factors: A global analysis. Environmental Research. 2020; 110355. doi: 10.1016/j.envres.2020.110355 33127399 PMC7591297

[18] Azuma K, Kagi N, Kim H, Hayashi M. Impact of climate and ambient air pollution on the epidemic growth during COVID-19 outbreak in Japan. Environmental Research. 2020;190: 110042. doi: 10.1016/j.envres.2020.110042 32800895 PMC7420955

[19] Raza A, Khan MTI, Ali Q, Hussain T, Narjis S. Association between meteorological indicators and COVID-19 pandemic in Pakistan. Environ Sci Pollut Res. 2020 [cited 8 Nov 2020]. doi: 10.1007/s11356-020-11203-2 33052566 PMC7556579

[20] Singh O, Bhardwaj P, Kumar D. Association between climatic variables and COVID-19 pandemic in National Capital Territory of Delhi, India. Environ Dev Sustain. 2020 [cited 8 Nov 2020]. doi: 10.1007/s10668-020-01003-6 33041646 PMC7538367

[21] Adhikari A, Yin J. Short-Term Effects of Ambient Ozone, PM2.5, and Meteorological Factors on COVID-19 Confirmed Cases and Deaths in Queens, New York. IJERPH. 2020;17: 4047. doi: 10.3390/ijerph17114047 32517125 PMC7312351

[22] Yao Y, Pan J, Liu Z, Meng X, Wang W, Kan H, et al. No association of COVID-19 transmission with temperature or UV radiation in Chinese cities. Eur Respir J. 2020;55: 2000517. doi: 10.1183/13993003.00517-2020 32269084 PMC7144256

[23] Ran J, Zhao S, Han L, Liao G, Wang K, Wang MH, et al. A re-analysis in exploring the association between temperature and COVID-19 transmissibility: an ecological study with 154 Chinese cities. Eur Respir J. 2020;56: 2001253. doi: 10.1183/13993003.01253-2020 32631839 PMC7338403

[24] Morris DH, Yinda KC, Gamble A, Rossine FW, Huang Q, Bushmaker T, et al. Mechanistic theory predicts the effects of temperature and humidity on inactivation of SARS-CoV-2 and other enveloped viruses. eLife. 2021;10: e65902. doi: 10.7554/eLife.65902 33904403 PMC8277363

[25] Comunian S, Dongo D, Milani C, Palestini P. Air Pollution and COVID-19: The Role of Particulate Matter in the Spread and Increase of COVID-19’s Morbidity and Mortality. IJERPH. 2020;17: 4487. doi: 10.3390/ijerph17124487 32580440 PMC7345938

[26] Fattorini D, Regoli F. Role of the chronic air pollution levels in the Covid-19 outbreak risk in Italy. Environmental Pollution. 2020;264: 114732. doi: 10.1016/j.envpol.2020.114732 32387671 PMC7198142

[27] Zhang Z, Xue T, Jin X. Effects of meteorological conditions and air pollution on COVID-19 transmission: Evidence from 219 Chinese cities. Science of The Total Environment. 2020;741: 140244. doi: 10.1016/j.scitotenv.2020.140244 32592975 PMC7832158

[28] Wang B, Chen H, Chan YL, Oliver BG. Is there an association between the level of ambient air pollution and COVID-19? American Journal of Physiology-Lung Cellular and Molecular Physiology. 2020;319: L416–L421. doi: 10.1152/ajplung.00244.2020 32697597 PMC7839633

[29] Jiang Y, Wu X-J, Guan Y-J. Effect of ambient air pollutants and meteorological variables on COVID-19 incidence. Infect Control Hosp Epidemiol. 2020;41: 1011–1015. doi: 10.1017/ice.2020.222 32389157 PMC7298083

[30] Frontera A, Martin C, Vlachos K, Sgubin G. Regional air pollution persistence links to COVID-19 infection zoning. Journal of Infection. 2020;81: 318–356. doi: 10.1016/j.jinf.2020.03.045 32283151 PMC7151372

[31] Copiello S, Grillenzoni C. The spread of 2019-nCoV in China was primarily driven by population density. Comment on “Association between short-term exposure to air pollution and COVID-19 infection: Evidence from China” by Zhu et al. Science of The Total Environment. 2020;744: 141028. doi: 10.1016/j.scitotenv.2020.141028 32711328 PMC7365069

[32] Wei J-T, Liu Y-X, Zhu Y-C, Qian J, Ye R-Z, Li C-Y, et al. Impacts of transportation and meteorological factors on the transmission of COVID-19. International Journal of Hygiene and Environmental Health. 2020;230: 113610. doi: 10.1016/j.ijheh.2020.113610 32896785 PMC7448770

[33] Sobral MFF, Duarte GB, da Penha Sobral AIG, Marinho MLM, de Souza Melo A. Association between climate variables and global transmission oF SARS-CoV-2. Science of The Total Environment. 2020;729: 138997. doi: 10.1016/j.scitotenv.2020.138997 32353724 PMC7195330

[34] Rader B, Scarpino SV, Nande A, Hill AL, Adlam B, Reiner RC, et al. Crowding and the shape of COVID-19 epidemics. Nat Med. 2020 [cited 6 Nov 2020]. doi: 10.1038/s41591-020-1104-0 33020651

[35] Gargiulo C, Gaglione F, Guida C, Papa R, Zucaro F, Carpentieri G. The role of the urban settlement system in the spread of Covid-19 pandemic. The Italian case. TeMA—Journal of Land Use. 2020;Mobility and Environment: 189–212 Pages. doi: 10.6092/1970-9870/6864

[36] Gangemi S, Billeci L, Tonacci A. Rich at risk: socio-economic drivers of COVID-19 pandemic spread. Clin Mol Allergy. 2020;18: 12. doi: 10.1186/s12948-020-00127-4 32617078 PMC7327192

[37] Diop BZ, Ngom M, Pougué Biyong C, Pougué Biyong JN. The relatively young and rural population may limit the spread and severity of COVID-19 in Africa: a modelling study. BMJ Glob Health. 2020;5: e002699. doi: 10.1136/bmjgh-2020-002699 32451367 PMC7252974

[38] The World Bank. World Development Indicators. 2017. Available: https://databank.worldbank.org/source/world-development-indicators

[39] Geneva, World Health Organization. Global Health Estimates 2015: Deaths by Cause, Age, Sex, by Country and by Region, 2000–2015. 2016. Available: https://www.who.int/healthinfo/global_burden_disease/estimates_regional_2000_2015/en/

[40] Kreutz R, Algharably EAE-H, Azizi M, Dobrowolski P, Guzik T, Januszewicz A, et al. Hypertension, the renin–angiotensin system, and the risk of lower respiratory tract infections and lung injury: implications for COVID-19. Cardiovascular Research. 2020;116: 1688–1699. doi: 10.1093/cvr/cvaa097 32293003 PMC7184480

[41] Ghosh D, Bernstein JA, Mersha TB. COVID-19 pandemic: The African paradox. Journal of Global Health. 2020;10: 020348. doi: 10.7189/jogh.10.020348 33110546 PMC7506193

[42] Central Intelligence Agency. GINI Index. In: The World Factbook [Internet]. 2017. Available: https://www.cia.gov/library/publications/the-world-factbook/rankorder/2172rank.html

[43] Oronce CIA, Scannell CA, Kawachi I, Tsugawa Y. Association Between State-Level Income Inequality and COVID-19 Cases and Mortality in the USA. Journal of General Internal Medicine. 2020;35: 2791–2793. doi: 10.1007/s11606-020-05971-3 32583336 PMC7313247

[44] The World Bank. World Development Indicators. 2019. Available: https://databank.worldbank.org/source/world-development-indicators

[45] The World Bank. Climate Data API. 2011. Available: https://datahelpdesk.worldbank.org/knowledgebase/articles/902061-climate-data-api

[46] Nuclear Threat Initiative, Johns Hopkins Center for Health Security, The Economist Intelligence Unit. GHS Index. 2019. Available: https://www.ghsindex.org/

[47] V-Dem Institute. V-dem (version 10). 2020. Available: https://www.v-dem.net/

[48] Chan MS, Winneg K, Hawkins L, Farhadloo M, Jamieson KH, Albarracín D. Legacy and social media respectively influence risk perceptions and protective behaviors during emerging health threats: A multi-wave analysis of communications on Zika virus cases. Social Science & Medicine. 2018;212: 50–59. doi: 10.1016/j.socscimed.2018.07.007 30005224 PMC6093206

[49] Bavel JJV, Baicker K, Boggio PS, Capraro V, Cichocka A, Cikara M, et al. Using social and behavioural science to support COVID-19 pandemic response. Nat Hum Behav. 2020;4: 460–471. doi: 10.1038/s41562-020-0884-z 32355299

[50] Pequeno P, Mendel B, Rosa C, Bosholn M, Souza JL, Baccaro F, et al. Air transportation, population density and temperature predict the spread of COVID-19 in Brazil. PeerJ. 2020;8: e9322. doi: 10.7717/peerj.9322 32547889 PMC7275681

[51] Kong JD, Tekwa EW, Gignoux-Wolfsohn SA. Data and Code for: Social, economic, and environmental factors influencing the basic reproduction number of COVID-19 across countries. 2020. Available: https://github.com/Jdkong/COVID-19 10.1371/journal.pone.0252373 PMC8189449 34106993

[52] Wallinga J, Lipsitch M. How generation intervals shape the relationship between growth rates and reproductive numbers. Proc R Soc B. 2007;274: 599–604. doi: 10.1098/rspb.2006.3754 17476782 PMC1766383

[53] Du Z, Xu X, Wu Y, Wang L, Cowling BJ, Meyers LA. Serial Interval of COVID-19 among Publicly Reported Confirmed Cases. Emerg Infect Dis. 2020;26. doi: 10.3201/eid2606.200357 32191173 PMC7258488

[54] Park M, Cook AR, Lim JT, Sun Y, Dickens BL. A Systematic Review of COVID-19 Epidemiology Based on Current Evidence. JCM. 2020;9: 967. doi: 10.3390/jcm9040967 32244365 PMC7231098

[55] He X, Lau EHY, Wu P, Deng X, Wang J, Hao X, et al. Temporal dynamics in viral shedding and transmissibility of COVID-19. Nat Med. 2020 [cited 7 May 2020]. doi: 10.1038/s41591-020-0869-5 32296168

[56] Wu K, Darcet D, Wang Q, Sornette D. Generalized logistic growth modeling of the COVID-19 outbreak: comparing the dynamics in the 29 provinces in China and in the rest of the world. Nonlinear Dyn. 2020;101: 1561–1581. doi: 10.1007/s11071-020-05862-6 32836822 PMC7437112

[57] Duhon J, Bragazzi N, Kong JD. The impact of non-pharmaceutical interventions, demographic, social, and climatic factors on the initial growth rate of COVID-19: A cross-country study. Science of The Total Environment. 2021;760: 144325. doi: 10.1016/j.scitotenv.2020.144325 33338848 PMC7728414

[58] Kissler SM, Tedijanto C, Goldstein E, Grad YH, Lipsitch M. Projecting the transmission dynamics of SARS-CoV-2 through the postpandemic period. Science. 2020;368: 860–868. doi: 10.1126/science.abb5793 32291278 PMC7164482

[59] Tuite AR, Fisman DN, Greer AL. Mathematical modelling of COVID-19 transmission and mitigation strategies in the population of Ontario, Canada. CMAJ. 2020;192: E497–E505. doi: 10.1503/cmaj.200476 32269018 PMC7234271

[60] Matrajt L, Leung T. Evaluating the Effectiveness of Social Distancing Interventions to Delay or Flatten the Epidemic Curve of Coronavirus Disease. Emerg Infect Dis. 2020;26: 1740–1748. doi: 10.3201/eid2608.201093 32343222 PMC7392458

[61] European Centre for Disease Prevention and Control. Download today’s data on the geographic distribution of COVID-19 cases worldwide. 2020. Available: https://www.ecdc.europa.eu/en/publications-data/download-todays-data-geographic-distribution-covid-19-cases-worldwide

[62] Roser M, Ritchie H, Ortiz-Ospina E, Hasell J. Coronavirus Disease (COVID-19). In: OurWorldInData.org [Internet]. 2020. Available: https://ourworldindata.org/coronavirus

[63] Russell TW, Golding N, Hellewell J, Abbott S, Wright L, Pearson CAB, et al. Reconstructing the early global dynamics of under-ascertained COVID-19 cases and infections. BMC Med. 2020;18: 332. doi: 10.1186/s12916-020-01790-9 33087179 PMC7577796

[64] Niehus R, De Salazar PM, Taylor A, Lipsitch M. Quantifying bias of COVID-19 prevalence and severity estimates in Wuhan, China that depend on reported cases in international travelers. Infectious Diseases (except HIV/AIDS); 2020 Feb. doi: 10.1101/2020.02.13.20022707 32511442 PMC7239063

[65] Zhao S, Lin Q, Ran J, Musa SS, Yang G, Wang W, et al. Preliminary estimation of the basic reproduction number of novel coronavirus (2019-nCoV) in China, from 2019 to 2020: A data-driven analysis in the early phase of the outbreak. International Journal of Infectious Diseases. 2020;92: 214–217. doi: 10.1016/j.ijid.2020.01.050 32007643 PMC7110798

[66] Russell TW, Hellewell J, Jarvis CI, van Zandvoort K, Abbott S, Ratnayake R, et al. Estimating the infection and case fatality ratio for coronavirus disease (COVID-19) using age-adjusted data from the outbreak on the Diamond Princess cruise ship, February 2020. Eurosurveillance. 2020;25. doi: 10.2807/1560-7917.ES.2020.25.12.2000256 32234121 PMC7118348

[67] Islam MS, Sarkar T, Khan SH, Mostofa Kamal A-H, Hasan SMM, Kabir A, et al. COVID-19–Related Infodemic and Its Impact on Public Health: A Global Social Media Analysis. The American Journal of Tropical Medicine and Hygiene. 2020;103: 1621–1629. doi: 10.4269/ajtmh.20-0812 32783794 PMC7543839

[68] Vosoughi S, Roy D, Aral S. The spread of true and false news online. Science. 2018;359: 1146–1151. doi: 10.1126/science.aap9559 29590045

[69] Sooknanan J, Comissiong DMG. Trending on Social Media: Integrating Social Media into Infectious Disease Dynamics. Bull Math Biol. 2020;82: 86. doi: 10.1007/s11538-020-00757-4 32617673 PMC7329999

[70] Boehmer TK, DeVies J, Caruso E, van Santen KL, Tang S, Black CL, et al. Changing Age Distribution of the COVID-19 Pandemic—United States, May–August 2020. MMWR Morb Mortal Wkly Rep. 2020;69: 1404–1409. doi: 10.15585/mmwr.mm6939e1 33001872 PMC7537561

[71] Dowd JB, Andriano L, Brazel DM, Rotondi V, Block P, Ding X, et al. Demographic science aids in understanding the spread and fatality rates of COVID-19. Proc Natl Acad Sci USA. 2020;117: 9696–9698. doi: 10.1073/pnas.2004911117 32300018 PMC7211934

[72] CMMID COVID-19 working group, Davies NG, Klepac P, Liu Y, Prem K, Jit M, et al. Age-dependent effects in the transmission and control of COVID-19 epidemics. Nat Med. 2020;26: 1205–1211. doi: 10.1038/s41591-020-0962-9 32546824

[73] Cowling BJ, Ali ST, Ng TWY, Tsang TK, Li JCM, Fong MW, et al. Impact assessment of non-pharmaceutical interventions against coronavirus disease 2019 and influenza in Hong Kong: an observational study. The Lancet Public Health. 2020;5: e279–e288. doi: 10.1016/S2468-2667(20)30090-6 32311320 PMC7164922

[74] Krkošek M, Jarvis-Cross M, Wadhawan K, Berry I, Soucy J-PR, Bodner K, et al. Establishment, contagiousness, and initial spread of SARS-CoV-2 in Canada. Moher D, editor. FACETS. 2021;6: 180–194. doi: 10.1139/facets-2020-0055

[75] Hilton J, Keeling MJ. Estimation of country-level basic reproductive ratios for novel Coronavirus (SARS-CoV-2/COVID-19) using synthetic contact matrices. Flegg JA, editor. PLoS Comput Biol. 2020;16: e1008031. doi: 10.1371/journal.pcbi.1008031 32614817 PMC7363110

[76] Davies NG, Abbott S, Barnard RC, Jarvis CI, Kucharski AJ, Munday JD, et al. Estimated transmissibility and impact of SARS-CoV-2 lineage B.1.1.7 in England. Science. 2021;372: eabg3055. doi: 10.1126/science.abg3055 33658326 PMC8128288

