# Supplementary material for: Social, economic, and environmental factors influencing the basic reproduction number of COVID-19 across countries
Source: PLoS One. 2021 Jun 9;16(6):e0252373. doi: 10.1371/journal.pone.0252373 (PMC8189449; doi:10.1371/journal.pone.0252373)
Supplement: S1 Fig — Dots represent daily cases averaged over a 7-day window, and curves are fitted based on the logistic growth model. (DOCX) [file pone.0252373.s001.docx]

**Supplementary Information for**

**Social, economic, and environmental factors influencing the basic reproduction number of COVID-19 across countries**

Jude Dzevela Kong^1,2,+^, Edward W. Tekwa^3,4,5,+^, Sarah A. Gignoux-Wolfsohn^6^

^1^Centre for Diseases Modeling (CDM), York University, Toronto, ON M3J 1P3, Canada

^2^Department of Mathematics and Statistics, York University, Toronto, ON M3J 1P3, Canada

^3^Department of Ecology, Evolution, and Natural Resources, Rutgers University, New Brunswick, NJ 08901, USA

^4^Department of Ecology and Evolutionary Biology, Princeton University, Princeton, NJ 08544-1003, USA

^5^Department of Ecology and Evolutionary Biology, University of Toronto, Toronto, ON M5S 3B2, Canada

^6^Smithsonian Environmental Research Center, Edgewater, MD 21037, USA

^+^Co-first authors

*Corresponding author: Jude Kong

**Email:** jdkong@yorku.ca

**Keywords:** COVID-19, basic reproduction number, social media, socio-demographic, environment

**
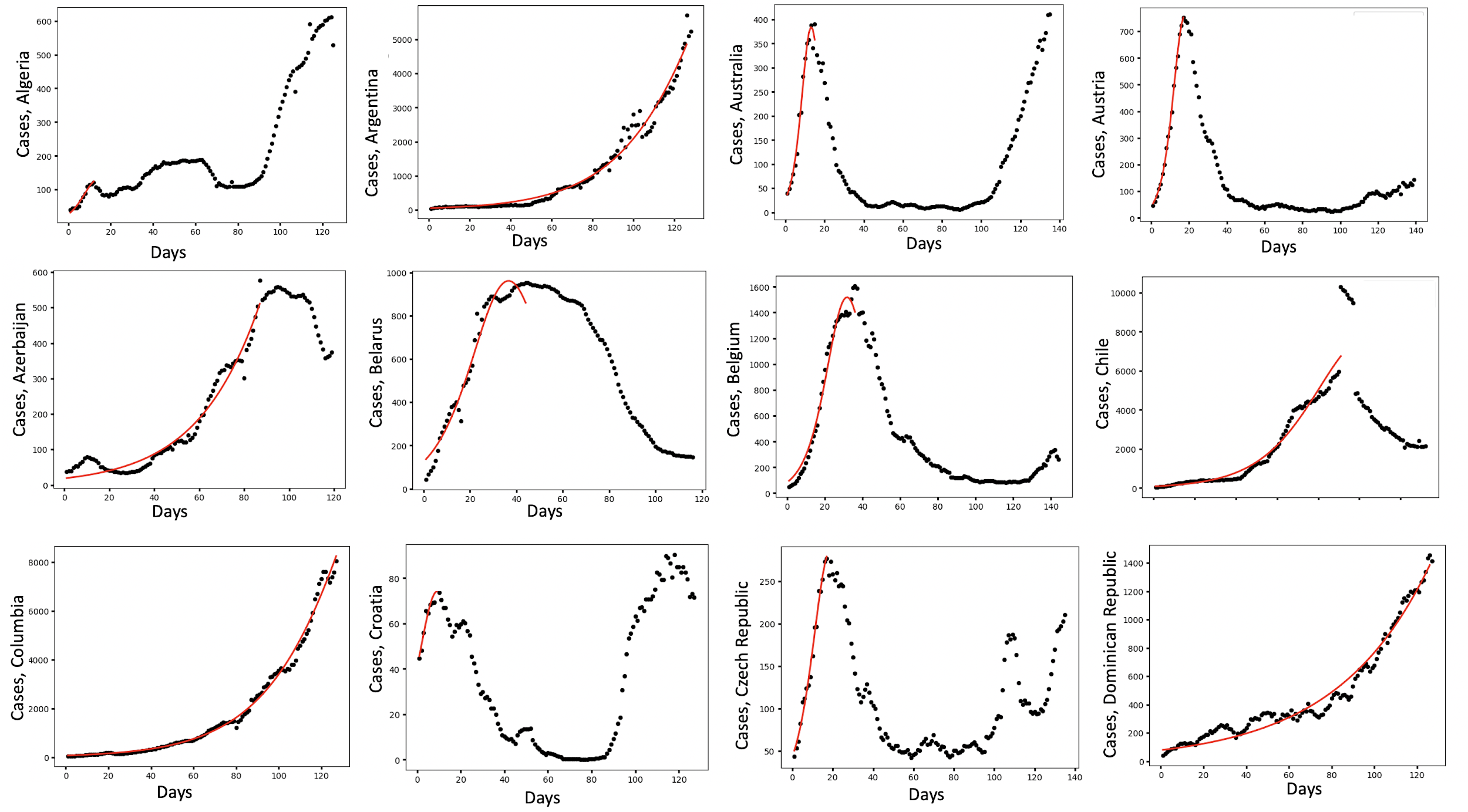
**

**Fig. S1. The COVID-19 daily cases.** Dots represent daily cases averaged over a 7-day window, and curves are fitted based on the logistic growth model.
