# Supplementary material for: Social, economic, and environmental factors influencing the basic reproduction number of COVID-19 across countries
Source: PLoS One. 2021 Jun 9;16(6):e0252373. doi: 10.1371/journal.pone.0252373 (PMC8189449; doi:10.1371/journal.pone.0252373)
Supplement: S3 Fig — Dots represent daily cases averaged over a 7-day window, and curves are fitted based on the logistic growth model. (DOCX) [file pone.0252373.s003.docx]

**
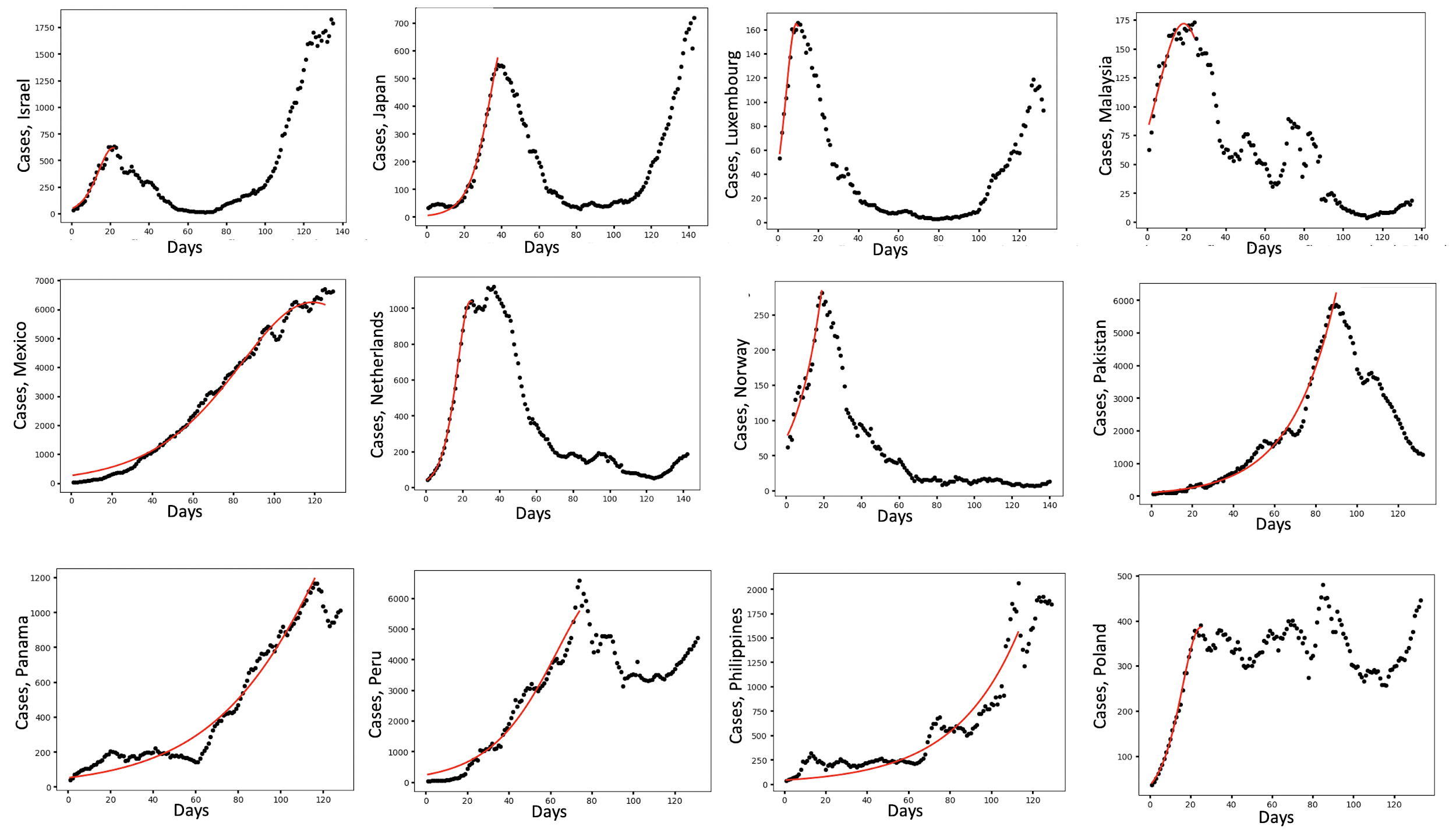
**

**Fig. S3. The COVID-19 daily cases.** Dots represent daily cases averaged over a 7-day window, and curves are fitted based on the logistic growth model.
