# Supplementary material for: Social, economic, and environmental factors influencing the basic reproduction number of COVID-19 across countries
Source: PLoS One. 2021 Jun 9;16(6):e0252373. doi: 10.1371/journal.pone.0252373 (PMC8189449; doi:10.1371/journal.pone.0252373)
Supplement: S5 Fig — Circles are partial residuals, and red shades are 95% confidence intervals. (DOCX) [file pone.0252373.s005.docx]

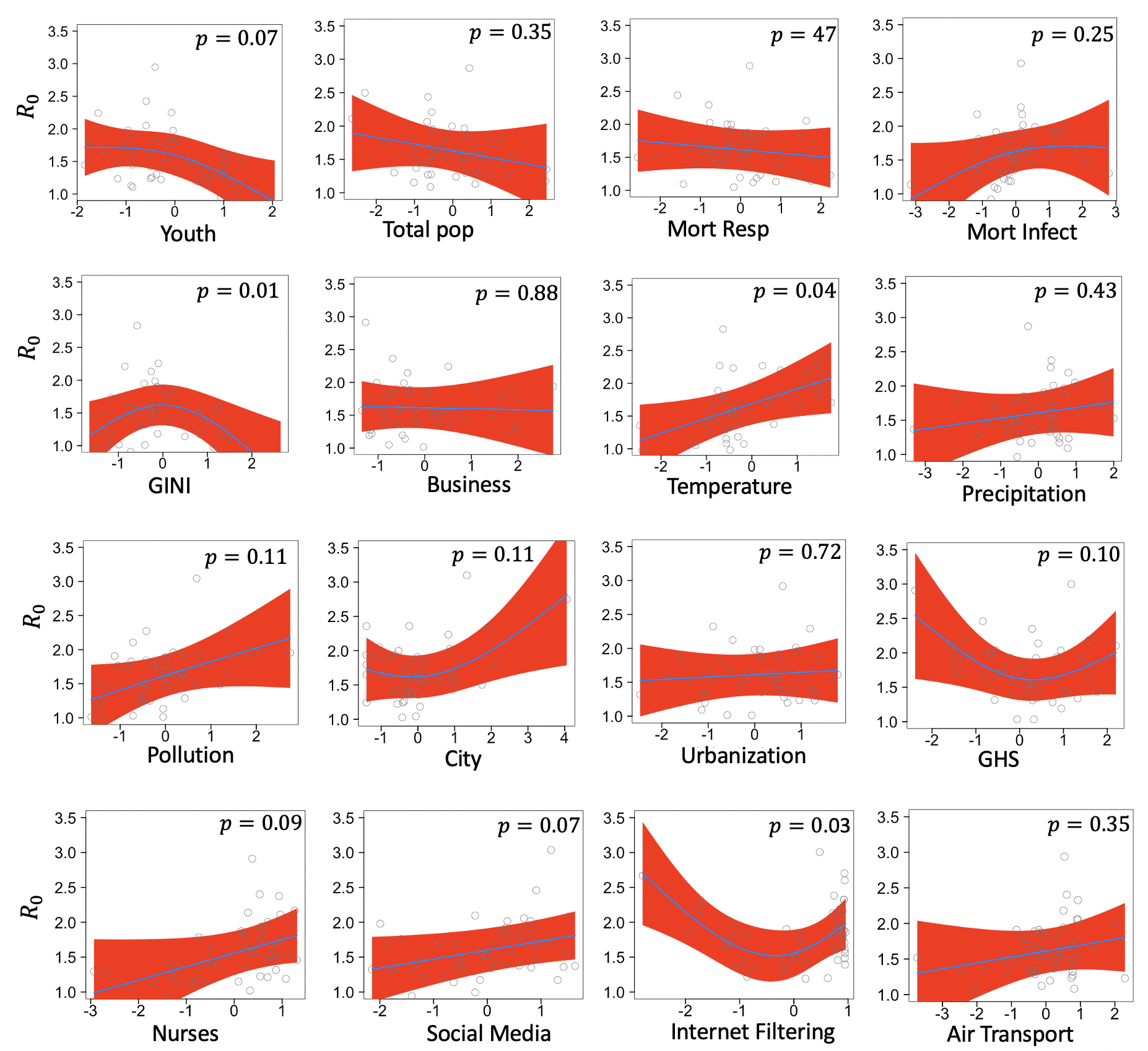


**Fig. S5. Fixed GAM derived effects of covariates on *R_0_* across countries.** Circles are partial residuals, and red shades are 95% confidence intervals.
