# Supplementary material for: Social, economic, and environmental factors influencing the basic reproduction number of COVID-19 across countries
Source: PLoS One. 2021 Jun 9;16(6):e0252373. doi: 10.1371/journal.pone.0252373 (PMC8189449; doi:10.1371/journal.pone.0252373)
Supplement: S1 Table — (DOCX) [file pone.0252373.s007.docx]

**Table S1. COVID-19 intrinsic growth rates and *R_0_*.**

| **Country** | **Growth rate** | ***R_0_*** |
| --- | --- | --- |
| Algeria | 0.20 | 2.26 |
| Argentina | 0.04 | 1.17 |
| Australia | 0.30 | 3.35 |
| Austria | 0.24 | 2.59 |
| Azerbaijan | 0.04 | 1.17 |
| Belarus | 0.09 | 1.44 |
| Belgium | 0.13 | 1.71 |
| Brazil | 0.04 | 1.18 |
| Canada | 0.08 | 1.38 |
| Chile | 0.06 | 1.25 |
| China | 0.17 | 2.00 |
| Colombia | 0.04 | 1.17 |
| Croatia | 0.18 | 2.02 |
| Czech Republic | 0.17 | 1.96 |
| Dominican Republic | 0.02 | 1.10 |
| Ecuador | 0.12 | 1.59 |
| Egypt | 0.06 | 1.29 |
| Estonia | 0.07 | 1.30 |
| Finland | 0.06 | 1.25 |
| France | 0.15 | 1.85 |
| Germany | 0.18 | 2.07 |
| Ghana | 0.02 | 1.10 |
| Greece | 0.05 | 1.23 |
| Hungary | 0.08 | 1.35 |
| Iceland | 0.14 | 1.72 |
| India | 0.03 | 1.15 |
| Indonesia | 0.03 | 1.12 |
| Iran | 0.07 | 1.31 |
| Ireland | 0.09 | 1.41 |
| Israel | 0.18 | 2.09 |
| Italy | 0.18 | 2.04 |
| Japan | 0.15 | 1.79 |
| Luxemburg | 0.28 | 3.01 |
| Malaysia | 0.10 | 1.50 |
| Mexico | 0.04 | 1.16 |
| Netherland | 0.20 | 2.19 |
| Norway | 0.07 | 1.33 |
| Pakistan | 0.05 | 1.20 |
| Panama | 0.03 | 1.13 |
| Peru | 0.05 | 1.24 |
| Philippines | 0.03 | 1.14 |
| Poland | 0.15 | 1.81 |
| Portugal | 0.23 | 2.47 |
| Romania | 0.15 | 1.86 |
| Russia | 0.10 | 1.47 |
| Serbia | 0.11 | 1.56 |
| Singapore | 0.23 | 2.47 |
| Slovenia | 0.03 | 1.15 |
| South Korea | 0.31 | 3.52 |
| Spain | 0.21 | 2.32 |
| Sweden | 0.09 | 1.41 |
| Switzerland | 0.20 | 2.18 |
| Thailand | 0.19 | 2.10 |
| Turkey | 0.16 | 1.89 |
| Ukraine | 0.15 | 1.80 |
| United Kingdom | 0.15 | 1.83 |
| United States | 0.18 | 2.07 |
| Uzbekistan | 0.05 | 1.21 |
