# Supplementary material for: Social, economic, and environmental factors influencing the basic reproduction number of COVID-19 across countries
Source: PLoS One. 2021 Jun 9;16(6):e0252373. doi: 10.1371/journal.pone.0252373 (PMC8189449; doi:10.1371/journal.pone.0252373)
Supplement: S2 Table — An edf of 1 is equivalent to a straight line. An edf of 2 is equivalent to a quadratic curve, and so on, with higher edfs describing more wiggly curves. (DOCX) [file pone.0252373.s008.docx]

**Table S2. Mixed effect GAM results.** An edf of 1 is equivalent to a straight line. An edf of 2 is equivalent to a quadratic curve, and so on, with higher edfs describing more wiggly curves.

| Covariate | Effective degrees of freedom (edf). | Reference degrees of freedom (Ref.df) | F-statistic | p-value |
| --- | --- | --- | --- | --- |
| Youth | 1.862 | 1.978 | 2.590 | 0.07179 |
| Total Pop | 1 | 1 | 0.001 | 0.97483 |
| Mort Resp | 1 | 1 | 0.394 | 0.53454 |
| Mort Infect | 1.77 | 1.944 | 2.105 | 0.16691 |
| GINI | 1.904 | 1.989 | 4.320 | 0.01826 |
| Business | 1 | 1 | 0.328 | 0.57036 |
| Temperature | 1 | 1 | 1.519 | 0.22596 |
| Precipitation | 1 | 1 | 1.622 | 0.21127 |
| Pollution | 1 | 1 | 2.460 | 0.12576 |
| City | 1.855 | 1.978 | 3.540 | 0.03069 |
| Urbanization | 1 | 1 | 0.587 | 0.44885 |
| GHS | 1.784 | 1.951 | 2.130 | 0.16376 |
| Nurses | 1 | 1 | 1.831 | 0.18465 |
| Social Media | 1 | 1 | 5.059 | 0.03084 |
| Internet Filtering | 1.693 | 1.888 | 1.521 | 0.29431 |
| Air Transport | 1 | 1 | 1.051 | 0.31226 |
| GDP | 1.440e-12 | 1 | 0.000 | 0.27051 |
| # of data points | 8.711e-01 | 1 | 8.697 | 0.00333 |
| # of days to first 30 cases | 7.069e-01 | 1 | 4.356 | 0.01421 |
| Region | 6.491e-11 | 7 | 0.000 | 0.68376 |
| Under-reported % | 2.250e-11 | 1 | 0.000 | 0.90051 |

R-sq.(adj) = 0.593 Deviance explained = 75.3%

GCV = 0.21806 Scale est. = 0.12991 n = 58
