# Supplementary material for: Social, economic, and environmental factors influencing the basic reproduction number of COVID-19 across countries
Source: PLoS One. 2021 Jun 9;16(6):e0252373. doi: 10.1371/journal.pone.0252373 (PMC8189449; doi:10.1371/journal.pone.0252373)
Supplement: S3 Table — An edf of 1 is equivalent to a straight line. An edf of 2 is equivalent to a quadratic curve, and so on, with higher edfs describing more wiggly curves. (DOCX) [file pone.0252373.s009.docx]

**Table S3. Fixed effect GAM results.** An edf of 1 is equivalent to a straight line. An edf of 2 is equivalent to a quadratic curve, and so on, with higher edfs describing more wiggly curves.

| Covariate | Effective degrees of freedom (edf). | Reference degrees of freedom (Ref.df) | F-statistic | p-value |
| --- | --- | --- | --- | --- |
| Youth | 1.706 | 1.906 | 2.549 | 0.0696 |
| Total Pop | 1 | 1 | 0.879 | 0.3546 |
| Mort Resp | 1 | 1 | 0.532 | 0.4705 |
| Mort Infect | 1.646 | 1.866 | 1.821 | 0.2544 |
| GINI | 1.930 | 1.994 | 4.692 | 0.0138 |
| Business | 1 | 1 | 0.019 | 0.8899 |
| Temperature | 1 | 1 | 4.413 | 0.0426 |
| Precipitation | 1 | 1 | 0.643 | 0.4280 |
| Pollution | 1.073 | 1.138 | 2.860 | 0.1121 |
| City | 1.768 | 1.945 | 2.038 | 0.1094 |
| Urbanization | 1 | 1 | 0.129 | 0.7214 |
| GHS | 1.830 | 1.970 | 2.716 | 0.1015 |
| Nurses | 1 | 1 | 2.979 | 0.0928 |
| Social Media | 1 | 1 | 3.553 | 0.0674 |
| Internet Filtering | 1.934 | 1.995 | 4.088 | 0.0273 |
| Air Transport | 1 | 1 | 0.898 | 0.3496 |

R-sq.(adj) = 0.45 Deviance explained = 65.2%

GCV = 0.28193 Scale est. = 0.17553 n = 58
